# Acute gastroenteritis in primary care: a longitudinal study in the Swiss Sentinel Surveillance Network, Sentinella

**DOI:** 10.1007/s15010-017-1049-5

**Published:** 2017-08-04

**Authors:** Claudia Schmutz, Philipp Justus Bless, Daniel Mäusezahl, Marianne Jost, Mirjam Mäusezahl-Feuz, Swiss Sentinel Surveillance Network

**Affiliations:** 1 Swiss Tropical and Public Health Institute, Socinstrasse 57, 4002 Basel, Switzerland; 2 University of Basel, Petersplatz 1, 4001 Basel, Switzerland; 3 Federal Office of Public Health, Schwarzenburgstrasse 157, 3003 Bern, Switzerland

**Keywords:** Acute gastroenteritis, Sentinel surveillance, Primary health care, Switzerland, Antibiotics, Infectious intestinal diseases

## Abstract

**Purpose:**

Acute gastroenteritis (AG) leads to considerable burden of disease, health care costs and socio-economic impact worldwide. We assessed the frequency of medical consultations and work absenteeism due to AG at primary care level, and physicians’ case management using the Swiss Sentinel Surveillance Network “Sentinella”.

**Methods:**

During the 1-year, longitudinal study in 2014, 172 physicians participating in “Sentinella” reported consultations due to AG including information on clinical presentation, stool diagnostics, treatment, and work absenteeism.

**Results:**

An incidence of 2146 first consultations due to AG at primary care level per 100,000 inhabitants in Switzerland was calculated for 2014 based on reported 3.9 thousand cases. Physicians classified patients’ general condition at first consultation with a median score of 7 (1 = poor, 10 = good). The majority (92%) of patients received dietary recommendations and/or medical prescriptions; antibiotics were prescribed in 8.5%. Stool testing was initiated in 12.3% of cases; more frequently in patients reporting recent travel. Among employees (15–64 years), 86.3% were on sick leave. Median duration of sick leave was 4 days.

**Conclusions:**

The burden of AG in primary care is high and comparable with that of influenza-like illness (ILI) in Switzerland. Work absenteeism is substantial, leading to considerable socio-economic impact. Mandatory infectious disease surveillance underestimates the burden of AG considering that stool testing is not conducted routinely. While a national strategy to reduce the burden of ILI exists, similar comprehensive prevention efforts should be considered for AG.

**Electronic supplementary material:**

The online version of this article (doi:10.1007/s15010-017-1049-5) contains supplementary material, which is available to authorized users.

## Background

Acute gastroenteritis (AG) is a common disease in humans worldwide. Case definition varies between studies and countries but mostly includes signs and symptoms of diarrhoea, vomiting, nausea, abdominal cramps or pain, fever, and blood or mucus in the stool [1–5]. AG can be caused by a wide variety of pathogens ranging from viruses and bacteria to protozoa and other parasites [5]. A study in Austria identified norovirus, *Clostridium difficile* and rotavirus as the most frequent aetiological agents in patients consulting general practitioners (GPs) due to AG [4]. Norovirus, rotavirus, sapovirus and *Campylobacter* spp. were the most common organisms among cases of infectious intestinal disease (IID) presenting to the GP in the UK [6].

Bacterial pathogens causing AG which have to be reported to the National Notification System for Infectious Diseases (NNSID) include positive laboratory tests for *Campylobacter* spp., *Salmonella* spp., and *Shigella* spp. as well as clinical and laboratory reports of positively tested patients with *Listeria monocytogenes* and enterohaemorrhagic *Escherichia coli* (EHEC). None of the above-mentioned viral causes of AG are notifiable in Switzerland [7]. As a result, the NNSID underestimates the true burden of AG because of non-notifiable pathogens causing AG. Additionally, not every patient suffering from AG presents to a physician (under-ascertainment) and, the physician does not always initiate stool diagnosis to investigate the aetiology of the illness (under-reporting) [8, 9]. Hence, what is seen in the Swiss mandatory notification system represents only an incomplete picture of the burden of disease due to AG. The determinants of under-ascertainment or under-reporting have been described for several countries but not for Switzerland: In the UK, it is estimated that every case of IID reported to national surveillance represents 9.5 cases presenting to a GP or 147 cases in the community [6]. In the Netherlands, 8% of patients with an IID visited a physician [10]. Van Cauteren et al. [11] estimated that of 115 community cases of campylobacteriosis and 20 community cases of salmonellosis one case is reported to the surveillance system in France. However, it has to be noted that the French surveillance systems are voluntary for these two pathogens.

Swiss routine surveillance data suggest an increasing frequency of campylobacteriosis and a decreasing frequency of salmonellosis [12]. More than half of campylobacteriosis patients in a case–control study approached a physician within 3 days after onset of symptoms and 14.5% were hospitalised [13]. A subsequent qualitative survey among primary care physicians described case management approaches including treatment strategies and stool diagnostic testing behaviours from the physicians’ perspective for patients with AG [8]. Four main approaches were identified of which only two—the “test & wait” and the “test & treat” approaches—include stool specimen testing and, hence, would result in case registration in the mandatory disease surveillance system in case of a positive test outcome. Healthcare costs for AG in Switzerland were estimated at €29–45 million annually [14].

In Switzerland, we lack data on under-ascertainment and under-reporting. Under-ascertainment refers to people not seeking healthcare and, hence, not being captured by the surveillance system as defined by Gibbons et al. [9]. Under-reporting is defined as people seeking healthcare but not being reported because of under-diagnosis—not diagnosing or misdiagnosing the infection or pathogen—or under-notification—failure to report positive diagnoses [9].

This study within the Swiss Sentinel Surveillance Network, Sentinella, aimed at understanding the lower levels of the burden of illness pyramid and addressing the incidence of AG in a broader context. Specifically, the study aimed at understanding determinants of under-diagnosis by (1) estimating the incidence and burden of AG seen at the primary care level, (2) describing the physicians’ case management (diagnostics, treatment) of AG patients and (3) estimating the work loss due to AG of cases presenting to a physician.

## Methods

A 1-year, longitudinal study in Sentinella, during the year 2014, was conducted asking physicians to report cases of AG on a weekly basis (later referred to as data from the “weekly questionnaire”). A questionnaire about disease characteristics, stool testing, and treating strategies was completed for a subset of cases (later referred to as “supplementary questionnaire”).

### Study setting

Sentinella is a voluntary surveillance system and research network of primary care physicians existing since 1986 which is operated and funded by the Federal Office of Public Health (FOPH). Physicians are organised in six geographical regions, each having its representative within the Sentinella steering committee. The steering committee, consisting of physicians and researchers of academic primary care institutes, meets regularly to set the research priorities and to decide on submitted projects. Our study was accepted to run in 2014.

During the Sentinella-year 2014, 172 physicians (47% general practitioners, 37% internists and 16% paediatricians; thereafter referred to as “Sentinella-physicians”) covering entire Switzerland were active in the network. In Switzerland, 6930 physicians were practicing in the ambulatory sector with the main specialty “general internal medicine” (summarising general practitioners and internists) or “paediatrics” in 2014 according to the Swiss medical association FMH [15]. Among these, 86% were practicing in general internal medicine and 14% in paediatrics.

### Case definition

A case of AG was defined as (a) a patient consulting a Sentinella-physician for the first time during the illness episode and suffering from diarrhoea (at least 3 watery or pasty stools daily; for at least 24 h but 14 days the longest) likely due to an infectious cause or (b) a patient consulting a Sentinella-physician for the first time during the illness episode with vomiting and abdominal cramps without significant diarrhoea, likely due to an infectious cause. Patients were excluded if diarrhoea was due to a known gastrointestinal disease (e.g. Crohn’s disease, ulcerative colitis, coeliac disease), medication intake (e.g. antibiotics) or food intolerance. Also patients with persistent diarrhoea (>14 days), or if vomiting was due to pregnancy, were excluded.

### Data collection

Sentinella-physicians reported basic data on patients suffering from AG on a weekly questionnaire, and more detailed data for a subsample of patients through a supplementary questionnaire which were available in German and French. German versions of the weekly (part on AG only) and supplementary questionnaires are available online (see Electronic Supplementary Material 1). The questionnaires were piloted with 10 general practitioners.

The weekly questionnaire included information on sex, date of birth, stool testing and hospitalisation of all AG patients (see case definition) seen in the corresponding week. The supplementary questionnaire contained additional questions on employment status, dates of symptom onset and consultation(s), signs and symptoms until first consultation, general condition, antibiotic and symptomatic treatment, stool testing, sick leave, hospitalisation, sequelae, and selected risk exposures in the 7 days preceding symptom onset.

Weekly questionnaires were available on paper and electronically according to the Sentinella standard procedure (method chosen by physician). More than half of the Sentinella-physicians reported electronically, all others reported on paper. Supplementary questionnaires were available on paper only. While weekly paper questionnaires were sent to the FOPH once a week by postal mail according to routine procedures, Sentinella-physicians were asked to send the supplementary questionnaire as soon as they considered the corresponding case as “completed”. Weekly electronic questionnaires were entered directly into the Sentinella-database by the Sentinella-physician.

Information available on Sentinella-physicians included the physicians’ specialty and location of practice. Sentinella-physicians additionally reported the total number of daily physician–patient contacts (PPCs) on the weekly questionnaire. A PPC is defined as each consultation independent of place (in practice or as domiciliary visit) and time (during or off consultation-hour or on emergency service) and serves as denominator for calculating disease incidence rates.

### Subsample for supplementary questionnaire

We expected that each Sentinella-physician would report around two AG cases per week based on the pilot testing and discussions with physicians. Assuming that 150 physicians report during 48 weeks, 14,400 cases were expected during the 1-year-study period. To reduce the anticipated work load for Sentinella-physicians but still reaching an appropriate sample size allowing for estimates with acceptable precision, we decided to apply the supplementary questionnaire to a subsample of cases. The targeted subsample size was set at 4800 cases (one-third of all cases). A sampling scheme was defined whereby every Sentinella-physician had to complete supplementary questionnaires during four consecutive weeks four times a year (=16 weeks per physician per year). We randomly assigned each Sentinella-physician a sampling pattern with sampling periods distributed equally over the year, hence not allowing for two consecutive sampling periods.

Case numbers in the first half of the study period were lower than expected necessitating the sampling scheme to change to full sampling. Starting from week 25 (starting on 14.06.2014), supplementary questionnaires had to be completed for every AG patient until the end of the study.

### Data entry and analysis

Weekly questionnaires on paper forms and all supplementary questionnaires were entered into the electronic Sentinella database at the FOPH. Ten percent of supplementary questionnaires was randomly selected for double entry to assess data quality. Double entries of questionnaires were compared and discrepancies were eliminated by re-checking against the original paper forms.

Cases of Sentinella-physicians who reported PPC for less than 75% of the weeks during the study period, i.e. <39 of 52 weeks were excluded from data analysis. This rule and cutoff value for regularly reporting physicians are standard for analyses of Sentinella data. Additionally, cases not fulfilling the case definition or cases where the Sentinella-physician spontaneously indicated a final diagnosis not in agreement with infectious AG were excluded from the analysis of supplementary questionnaire data.

Data of weekly questionnaires were analysed descriptively. We calculated the average number of cases per Sentinella-physician and week and the number of initial consultations due to AG per 1000 PPCs per week. Additionally, we estimated the incidence and total number of first consultations due to AG at the primary care level for 2014 in Switzerland by the standard extrapolation of the Sentinella system which is described elsewhere [16].

Due to the mid-study change in the sampling scheme of supplementary questionnaires, analyses of the supplementary questionnaire data were weighted according to the sampling probability: information from the supplementary questionnaire of cases reported during the first half of the study period was analysed using a sampling weight of 3.25 (as each physician was required to submit a supplementary questionnaire for each case seen during 16 of 52 weeks; 1/(16/52) = 3.25) while information reported during the second half had a sampling weight of 1 (supplementary questionnaire required for every case). Point-estimates including 95% confidence intervals (CI) and interquartile ranges (IQR) for medians are reported for weighted analyses. Data from supplementary questionnaires were analysed descriptively and differences were assessed for significance by weighted, univariable logistic regression. For all analyses involving employment status, only patients aged 15–64 years were considered. Data were analysed and represented graphically using Stata 13.1 (StataCorp.). Maps were created using ArcGIS 10.2.1 for desktop (Environmental Systems Research Institute, Inc., Esri).

## Results

### Physician and patient characteristics

**Fig. 1:**
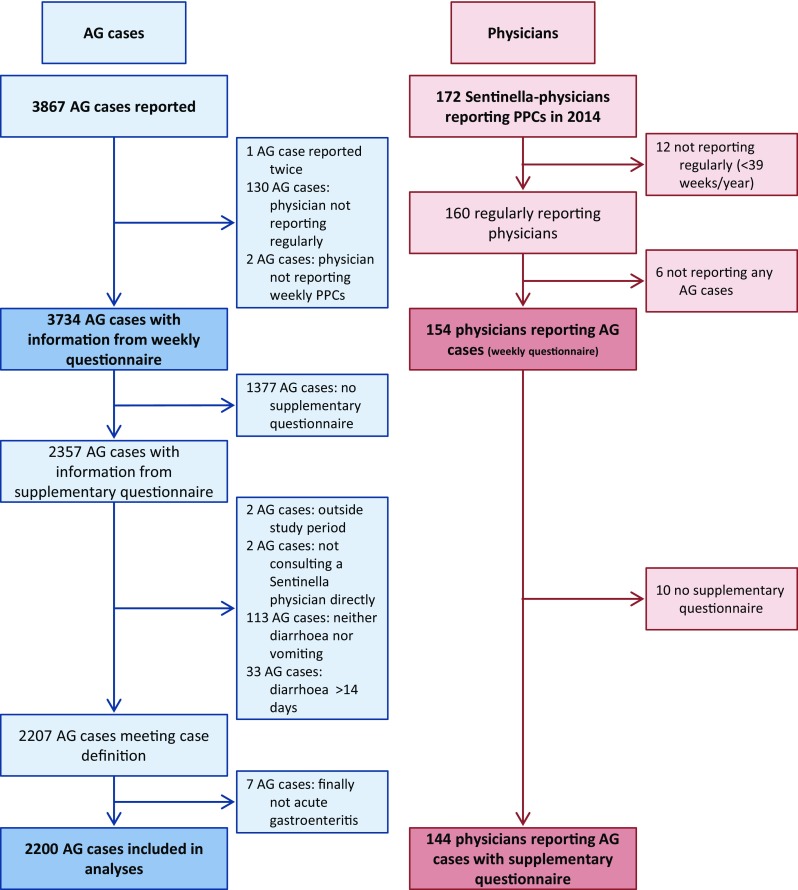
Study profile of notified cases and reporting physicians. Acute gastroenteritis study, Swiss Sentinel Surveillance Network, 2014. *AG* acute gastroenteritis, *PPC* physician–patient contact

In total, 3867 cases of AG were reported on weekly questionnaires by 172 participating Sentinella-physicians. After exclusion of cases reported by not regularly reporting Sentinella-physicians (130 cases) and for other reasons (3 cases), 3734 cases were used for analyses of weekly questionnaires. 2200 cases were retained for the analyses of supplementary questionnaires. The detailed inclusion process is described in Fig. 1.

Out of 172 physicians registered in the Sentinella system in 2014, 154 of the regularly reporting physicians reported at least one case of AG on the weekly questionnaire. Over the whole study period, individual physicians reported up to 400 cases (median 17, IQR 7–29). A total of 144 physicians submitted at least one supplementary questionnaire of a case fulfilling the case definition (Fig. 1). The subsample of cases with supplementary questionnaires was comparable to cases reported on weekly forms in terms of basic patient characteristics (Table 1).

**Table 1:** Basic characteristics of acute gastroenteritis cases reported on the weekly and supplementary questionnaires by physicians from the Swiss Sentinel Surveillance Network in 2014

	Weekly form	Supplementary questionnaire
Cases included in analysis (*N*)	3734	2200
Proportion of male cases, % (95% CI)	50.2	50.6 (48.0–53.3)
Median age, years (IQR)	21 (5–41)	22 (6.0 [95% CI 2.6–9.4]–43.0 [95% CI 38.1–47.9])
Physicians’ area of specialisation		
General medicine, % (95% CI)	35.3	37.5 (29.9–45.8)
Internal medicine, % (95% CI)	26.7	27.6 (21.1–35.4)
Paediatrics, % (95% CI)	38.0	34.9 (25.7–45.3)
Stool testing initiated, % (95% CI)	10.9	12.3 (10.1–14.8)
Hospitalised, % (95% CI)	2.0	2.7 (1.9–3.7)

**Fig. 2:**
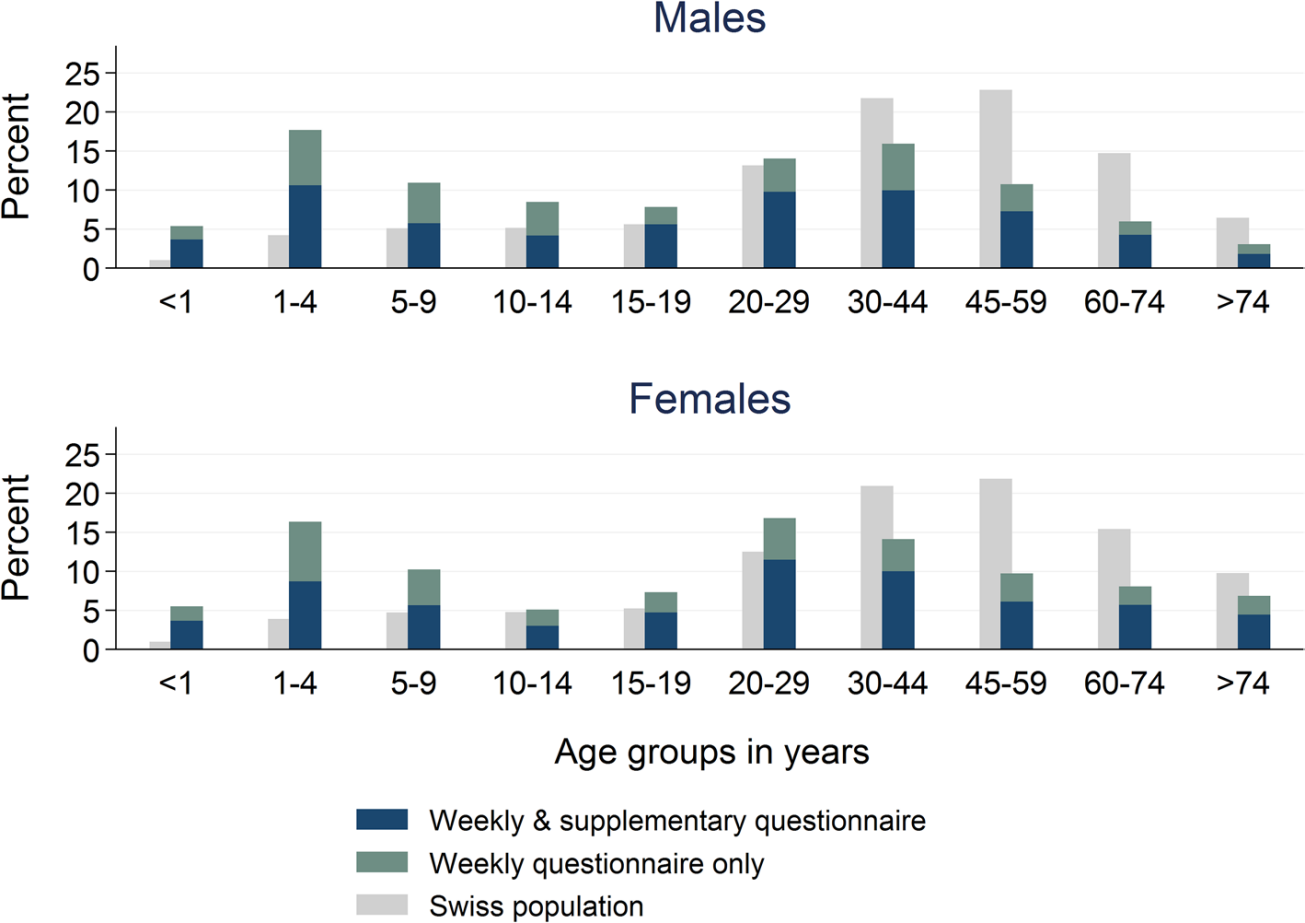
Age distribution by sex among acute gastroenteritis cases reported by Sentinella-physicians on weekly and/or supplementary questionnaires. Swiss Sentinel Surveillance Network, 2014; age distribution of Swiss population (official numbers [17]) added for comparison

Median age of AG cases was 21 years (IQR 5–41 years). Children, adolescents and young adults (age groups <1, 1–4, 5–9, 10–14, 15–19 and 20–29 years) were overrepresented among AG cases consulting a physician compared to the frequency of those age groups in the general Swiss population for both genders (Fig. 2). In the age group of 10–14 year olds, males were more frequent than females. In adults, female cases aged 20–29 years were most frequently reported while in males the 30–44 year age group predominated.

### Burden of AG at primary care level

**Fig. 3:**
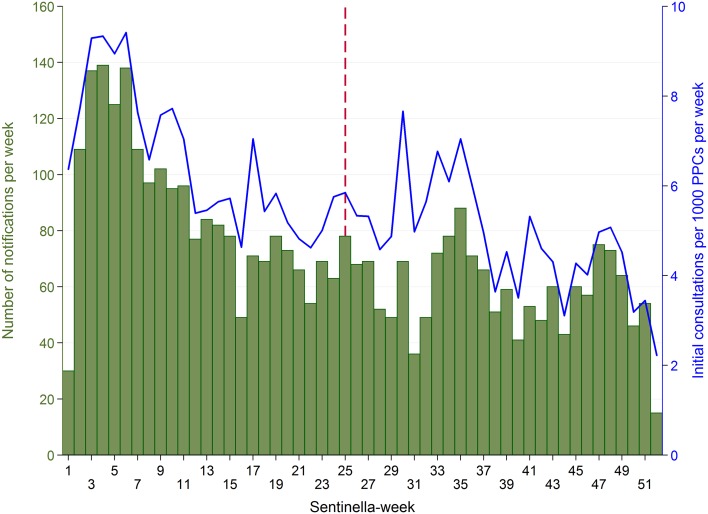
Acute gastroenteritis cases reported by physicians from the Swiss Sentinel Surveillance Network in 2014 (28.12.2013–26.12.2014): weekly case numbers (*bars*) and number of initial AG consultations per 1000 physician–patient contacts (PPCs, “consultations”) per week (*line*). *Vertical*, *dashed line* date of change of sampling scheme (from subsample of cases with supplementary questionnaires to supplementary questionnaire for every reported case)

**Fig. 4:**
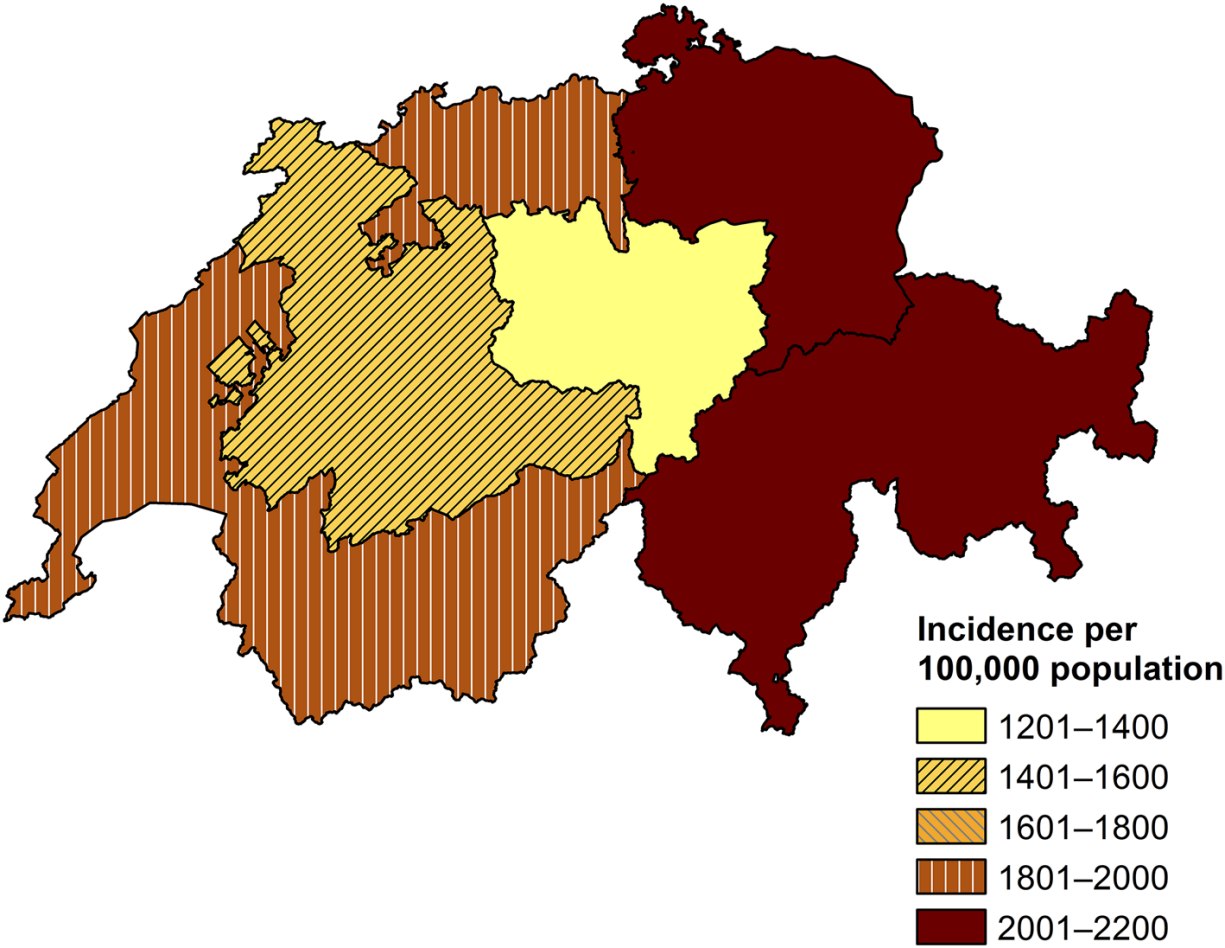
Calculated incidence of first consultations due to acute gastroenteritis at primary care level in Switzerland by Sentinella-region, based on standard extrapolation. Swiss Sentinel Surveillance Network, 2014. Note: an outlier (one physician reporting 400 cases) was excluded from this extrapolation by region. Source of map shapefile: Swiss Federal Office of Topography

Each week, 15–139 cases (median 69, IQR 54–80) were reported (Fig. 3). Case numbers were highest during the first weeks of the year (maximum in week 4) and decreased thereafter. A median rate of 5.4 first consultations due to AG per 1000 PPCs per week (IQR 4.6–6.7) was observed. The notifications correspond to 2146 first consultations due to AG at primary care level per 100,000 inhabitants or 174,610 first consultations due to AG in Switzerland in 2014 using the standard extrapolation method of the FOPH for Sentinella data. Incidence (of first consultations) by Sentinella-region is displayed in Fig. 4.

### Health care seeking and clinical presentation

The median time from symptom onset to first consultation was 2 days (95% CI 2.0–2.0, IQR 1.0 [95% CI 1.0–1.0]–3.0 [95% CI 2.4–3.6]). The majority of patients (87.9% [95% CI 85.6–89.9]) suffered from diarrhoea (Table 2). Loss of appetite was reported for 63.5% (95% CI 58.4–68.4), abdominal pain or cramps for 61.1% (95% CI 57.0–65.1), nausea for 60.4% (95% CI 56.6–64.1) and vomiting for 57.5% (95% CI 54.3–60.7) of patients. Less frequently reported signs and symptoms included flatulence, fever, dehydration and headache.

**Table 2:** Characteristics of cases with acute gastroenteritis at first consultation and number of consultations as reported by primary care physicians from the Swiss Sentinel Surveillance Network, 2014

	Number of cases [*n*]	Percent [%] (95% confidence interval)
Signs and symptoms until first consultation^a^ (*N* = 2200)		
Diarrhoea	1940	87.9 (85.6–89.9)
Diarrhoea with blood and/or mucus	249	10.8 (8.5–13.7)
Loss of appetite	1345	63.5 (58.4–68.4)
Abdominal pain/cramps	1329	61.1 (57.0–65.1)
Nausea	1296	60.4 (56.6–64.1)
Vomiting	1227	57.5 (54.3–60.7)
Flatulence	896	40.6 (35.6–45.7)
Fever	530	25.0 (22.3–27.9)
Dehydration	183	8.5 (6.6–11.0)
Headache	68	3.2 (2.1–4.8)
General condition at first consultation (according to physicians’ impression) (*N* = 2115)		
Poor: 1	1	0.09 (0.01–0.6)
2	28	1.1 (0.7–1.9)
3	95	4.6 (3.3–6.4)
4	177	8.4 (6.2–11.4)
5	237	10.7 (7.9–14.4)
6	228	10.1 (8.3–12.3)
7	318	15.8 (13.6–18.2)
8	476	23.9 (20.6–27.5)
9	356	16.5 (13.5–20.1)
Good: 10	199	8.7 (6.3–12.0)
Number of consultations (*N* = 2200)		
1	1742	79.6 (76.5–82.4)
2	365	16.4 (14.0–19.2)
3	75	3.2 (2.4–4.2)
4	18	0.8 (0.4–1.5)

^a^ Multiple answers possible

The majority of patients consulted the Sentinella-physician only once (79.6%, 95% CI 76.5–82.4) (Table 2). The median general condition of cases as reported by Sentinella-physicians at the time of first consultation was 7 (95% CI 6.5–7.5, IQR 5.0 [95% CI 4.5–5.5]–9.0 [95% CI 8.5–9.5]) on a rating scale from 1 (poor) to 10 (good). Overall, 86.3% (95% CI 83.1–89.0) of employed patients were unable to work. The odds for a good general condition (7 or above) was lower for employed patients compared to unemployed patients although not significantly (odds ratio [OR] 0.76, 95% CI 0.52–1.11, *p* = 0.159). The median duration of sick leave was 4 days (95% CI 3.8–4.2, IQR 3.0 [95% CI 3.0–3.0]–5.0 [95% CI 4.5–5.5]). For all except seven cases, the duration of sick leave was below 15 days.

The hospitalisation rate was 2.7% (95% CI 1.9–3.7). The highest hospitalisation rate was observed for the >74 year age group (11.5%, 95% CI 6.4–19.9) whereas for the remaining age groups the rates were below 4%. For 2.0% (95% CI 1.4–2.9) of patients, Sentinella-physicians reported sequelae, like dehydration, diverticulitis, or colitis. No deaths due to AG were reported.

### Stool diagnostics and results

Sentinella-physicians reported the initiation of stool specimen testing in 12.3% (95% CI 10.1–14.8); in 11.6% (95% CI 9.5–14.1) of cases they indicated that the sample was actually sent off (Table 3). The odds for stool testing did not differ between sexes ([female vs. male]: OR 1.13, 95% CI 0.84–1.50, *p* = 0.423) but differed by age group (*p* < 0.001): The proportion of stool testing was generally higher among older age groups. Paediatricians initiated stool testing less frequently (OR 0.32, 95% CI 0.18–0.55, *p* < 0.001) than general practitioners. The odds of initiating stool testing did not differ significantly for internists compared to general practitioners (OR 1.13, 95% CI 0.71–1.78, *p* = 0.610).

**Table 3:** Frequency of and reasons for prescription of stool diagnostics among acute gastroenteritis patients consulting primary care physicians from the Swiss Sentinel Surveillance Network, 2014

	Number of cases [*n*]	Percent [%] (95% confidence interval)
Stool test initiated (*N* = 2176)	286	12.3 (10.1–14.8)
Stool test performed (*N* = 2176)	272	11.6 (9.5–14.1)
Main reason for stool testing (*N* = 197)		
Protracted course of disease	62	29.4 (21.9–38.2)
Poor general condition	23	11.5 (6.9–18.4)
Specific symptom	19	9.5 (4.6–18.6)
Stay abroad before symptom onset	18	7.8 (4.5–13.1)
Comorbidity	10	5.3 (2.5–10.7)
Outbreak investigation	8	5.3 (1.6–16.4)
Occupation	10	3.8 (1.8–8.1)
Resident/patient institution	2	2.0 (0.5–8.0)
Age	2	1.3 (0.3–6.2)
Contact to animals	1	1.0 (0.1–6.8)
Contact to ill persons	1	0.3 (0.04–2.3)
Other reasons (e.g. elevated CRP level, leucocytosis, recent antibiotic therapy)	20	10.5 (6.5–16.6)
Reason not specified	21	12.2 (6.4–22.2)
Pathogens identified^a^ (*N* = 98)		
*Campylobacter* spp.	57	50.8 (39.2–62.3)
Norovirus	8	10.9 (5.0–21.9)
*Blastocystis* spp.	6	9.6 (4.0–21.1)
Rotavirus	5	8.9 (2.9–24.2)
*Clostridium* spp.	7	7.3 (2.9–17.2)
*Entamoeba* spp.	4	5.4 (1.7–15.8)
Pathogenic *E. coli*	6	5.3 (2.0–13.1)
*Candida* spp.	3	4.8 (1.4–15.6)
*Salmonella* spp.	6	3.8 (1.7–8.2)
Other (*Giardia* spp., adenovirus, *Aeromonas* spp., hepatitis E)	4	4.0 (1.2–12.5)

^a^ Two pathogens were identified in 11.5% (95% CI 5.4–22.9) of the 98 cases with a positive stool test result

Even though the questionnaire explicitly asked for the main reason for initiating stool testing, multiple answers were given for 31.0% (95% CI 24.9–37.8) of cases. The three most frequent reasons mentioned were protracted course of disease (29.4%, 95% CI 21.9–38.2), poor general condition (11.5%, 95% CI 6.9–18.4) and due to a specific symptom (9.5%, 95% CI 4.6–18.6) when excluding those with multiple answers. When considering also multiple answers, staying abroad before symptom onset was the third most frequent reason (data not shown).

Travelling within the 7 days preceding symptom onset was reported for 9.0% (95% CI 7.4–10.8) of cases. Patients with recent travel history were significantly more likely to undergo stool testing than patients not reporting any recent travels (OR 3.60, 95% CI 2.47–5.33, *p* < 0.001). Among patients with recent travel history, 30.0% (95% CI 22.7–38.6) were tested while for patients without travel to a foreign country in the 7 days preceding the symptom onset this proportion was 10.6% (95% CI 8.6–13.0). “Staying abroad” was indicated as the main reason for testing for 40.8% (95% CI 24.4–59.6) of patients with a travel history. Protracted course of disease was the second most often mentioned reason for stool testing among patients with travel history abroad (17.4%, 95% CI 7.2–36.2).

A positive test result was reported for more than one-third (35.9%, 95% CI 29.2–43.2) of tested patients while for the remaining 64.1% (95% CI 56.8–70.8) of patients test results were negative or not specified. The most frequently identified pathogen was *Campylobacter* spp. (50.8%, 95% CI 39.2–62.3) followed by norovirus (10.9%, 95% CI 5.0–21.9), and *Blastocystis* spp. (9.6%, 95% CI 4.0–21.1) (Table 3). Other pathogens identified included rotavirus, *Clostridium* spp., *Entamoeba* spp., pathogenic *E. coli*, *Candida* spp., *Salmonella* spp., *Giardia* spp., microsporidia, adenovirus, *Aeromonas* spp. and hepatitis E virus. Two pathogens were identified in 11.5% (95% CI 5.4–22.9) of the 98 cases with a positive stool test result.

### Approaches for symptomatic and antibiotic therapy

In 92.0% (95% CI 89.8–93.8) of cases, Sentinella-physicians gave dietary recommendations, or prescribed symptomatic and/or antibiotic treatment. Most commonly, patients were advised to care for fluid replacement by the intake of sufficient tea, broth etc. (58.3%, 95% CI 53.0–63.3) (Table 4). Distinct rehydration therapies such as electrolyte solution (11.4%, 95% CI 7.8–16.4) and infusion therapies (1.7%, 95% CI 1.1–2.6) were less frequently prescribed. Symptomatic treatment included probiotics (45.9%, 95% CI 39.1–52.8), antiemetics (45.4%, 95% CI 40.5–50.4), antidiarrhoeals (28.8%, 95% CI 23.6–34.6), analgesics (16.3%, 95% CI 12.8–20.5), and spasmolytics (15.0%, 95% CI 11.5–19.2). Antibiotics were prescribed in 8.5% (95% CI 6.5–11.0) of cases (Table 4).

**Table 4:** Frequency of prescription of antibiotic and symptomatic treatment, and reasons for prescription of antibiotic therapy among acute gastroenteritis patients consulting primary care physicians from the Swiss Sentinel Surveillance Network, 2014

	Number of cases [*n*]	Percent [%] (95% confidence interval)
Antibiotic therapy prescribed (*N* = 2089)	195	8.5 (6.5–11.0)
Antibiotic class prescribed^a^ (*N* = 195)		
Quinolone	123	60.2 (48.5–70.9)
Macrolide	30	15.0 (9.3–23.3)
Metronidazole	21	12.8 (7.7–20.5)
Aminopenicillin	22	11.6 (6.3–20.5)
Trimethoprim/sulfamethoxazole	7	4.5 (1.5–12.7)
Cephalosporin	5	3.1 (1.1–8.6)
Tetracycline	1	0.3 (0.0–2.4)
Not specified	5	1.6 (0.6–4.4)
Main reason for prescription of antibiotics (*N* = 195)		
Bacterial gastroenteritis	64	41.1 (25.0–59.5)
Duration of illness	12	9.0 (3.4–19.6)
Specific symptom	10	7.2 (3.4–14.8)
Expecting attitude of patient	6	4.5 (1.7–11.6)
Poor general condition	6	3.6 (1.3–9.2)
Immunosuppression	3	3.2 (0.9–11.0)
High, prolonged fever	5	3.1 (1.0–9.3)
Polymorbidity	4	2.7 (0.8–8.5)
Preventively	3	2.3 (0.6–8.5)
Other reasons (e.g. elevated CRP level, leucocytosis, co-infection)	22	13.3 (7.9–21.6)
Reason not specified	14	9.9 (5.2–18.2)
Recommended symptomatic treatment^a^ (*N* = 1909)		
Fluid replacement with tea, broth	1089	58.3 (53.0–63.3)
Probiotics	875	45.9 (39.1–52.8)
Antiemetics	851	45.4 (40.5–50.4)
Antidiarrhoeals	584	28.8 (23.6–34.6)
Analgesics	330	16.3 (12.8–20.5)
Spasmolytics	287	15.0 (11.5–19.2)
Rehydration solution	201	11.4 (7.8–16.4)
Intravenous rehydration	36	1.7 (1.1–2.6)

^a^ Multiple answers possible

The Sentinella-physicians initiated stool testing and prescribed antibiotics at the first consultation in 33 cases (unweighted results, Table 5). Stool diagnostics revealed the presence of a pathogen susceptible to antibiotics in 20 of these cases. No antibiotics were prescribed in 22 cases even though a pathogen which is theoretically susceptible to antibiotics was identified.

**Table 5:** Time point of prescription of stool testing and antibiotic treatment among acute gastroenteritis patients consulting primary care physicians, Swiss Sentinel Surveillance Network, 2014

	No antibiotics prescribed	Antibiotic prescribed at first consultation	Antibiotic prescribed at follow-up consultation
No stool test initiated	1713	70	11
Stool test initiated at first consultation	68	33	7
Thereof with positive result for a pathogen susceptible to antibiotic therapy^a^	12	20	5
Thereof with positive result for a pathogen not susceptible to antibiotic therapy^a^	4	1	
Stool test initiated at follow-up consultation	56	3	22
Thereof with positive result for a pathogen susceptible to antibiotic therapy^a^	10	2	11
Thereof with positive result for a pathogen not susceptible to antibiotic therapy^a^	4		1

Unweighted results. Cases with missing information on (date of) antibiotic prescription and/or (date of) stool test were excluded

^a^ Not considering possible antibiotic resistances and treatment recommendations

The majority of patients receiving antibiotics was treated with quinolones (60.2%, 95% CI 48.5–70.9), followed by macrolides, metronidazole, aminopenicillin, trimethoprim/sulfamethoxazole, cephalosporin and tetracycline (Table 4). Two or more antibiotic classes were reported to be used for 8.5% (95% CI 4.6–15.2) of cases. No antibiotic class was reported for 1.6% (95% CI 0.6–4.4) of cases treated with antibiotics.

Main reasons for the prescription of antibiotic therapy included (suspicion of) bacterial gastroenteritis (41.1%, 95% CI 25.0–59.5), duration of illness (9.0%, 95% CI 3.4–19.6), a specific symptom (7.2%, 95% CI 3.4–14.8) and others (Table 4). Sentinella-physicians mentioned several reasons for 23.9% (95% CI 16.6–32.2) of the patients despite being asked to indicate only the main reason. When considering also multiple answers, “poor general condition” was the third most frequently mentioned reason for antibiotic therapy (data not shown).

Similar to stool testing, antibiotic prescription was associated with age (*p* < 0.001) and with the physicians’ specialty (*p* < 0.001) but not with sex (*p* = 0.511) (data not shown). Again, children and adolescents were less frequently treated with antibiotics compared to adults. Among the >74-year-old age group, one-fifth of cases received antibiotics (20.0%, 95% CI 12.8–29.7). Nearly three-quarter of the antibiotic therapies were prescribed at the first consultation (71.3%, 95% CI 60.5–80.1). These patients had a lower general condition according to physicians’ impression (median 5.0, 95% CI 4.0–6.0, IQR 4.0 [95% CI 3.0–5.0]–7.0 [95% CI 6.0–8.0]) than patients receiving antibiotics later on (median 7.0, 95% CI 6.0–8.0, IQR 5.0 [95% CI 4.0–6.0]–8.0 [95% CI 7.0–9.0]) and also suffered slightly more frequently from fever (44.7%, 95% CI 34.5–55.4 vs. 38.9%, 95% CI 24.0–56.2). However, both differences were not statistically significant. Patients with a recent history of travel had significant higher odds to undergo antibiotic therapy (OR 1.75, 95% CI 1.06–2.88, *p* = 0.029).

## Discussion

This study underscored that acute gastroenteritis is common in Swiss primary care: extrapolated annual consultation numbers (175,000 first consultations) are comparable to those of influenza-like illness (ILI) during an influenza season (varying between 107,000 and 276,000 ILI cases in the last three seasons [18–20]). The majority of patients is symptomatically treated and does not require multiple consultations. However, most episodes of AG lead to a sick leave of several days, though the physician-assessed general state of the patients is considered as “fairly good”. Stool specimen testing is not systematically conducted and antibiotic therapy is applied to less than 10% of patients.

### Multiple factors influence physicians’ decision making

Sentinella-physicians reported more than one reason for stool testing in a third of cases despite being explicitly asked for the main reason in the questionnaire. This suggests that a combination of factors plays a role in decision making. The same holds true for the prescription of antibiotic treatment where in around a quarter of cases several reasons were mentioned albeit physicians were asked to indicate the main reason. The reasons mentioned most frequently for stool testing—namely protracted course of disease, poor general condition, due to a specific symptom and a history of recent travel—are in line with findings from other studies: three of the aforementioned four factors (all except “specific symptom”) were also mentioned by GPs participating in a qualitative study in Switzerland [8] and in a study from Northern Ireland and the Republic of Ireland [21]. The Irish study further reported that stool testing is frequently prescribed if the illness is associated with an outbreak or if the physicians suspect a link with a particular consumed food item or food premises (pub, restaurant, take away). Similarly, a qualitative study among GPs in the UK found that long duration of illness, recent travel, blood in the stool, patient being unwell and exclusion of an infectious cause were the reasons mentioned most frequently for stool testing [22]. Factors most strongly associated with requesting a stool culture were bloody diarrhoea, diarrhoea lasting more than 3 days, and a diagnosis of AIDS in a postal survey among physicians in the US [23].

Considering that protracted course of disease and poor general condition were mentioned most frequently as main reasons for stool testing in our study, the difference in reported general condition at the time of first consultation among tested and untested patients seems rather small (median 7.0, 95% CI 6.5–7.5, IQR 5.0 [95% CI 4.5–5.5]–8.0 [95% CI 7.5–8.5] vs. median 8.0, 95% CI 7.5–8.5, IQR 6.0 [95% CI 5.5–6.5]–9.0 [95% CI 8.5–9.5]). One explanation for this is that a “protracted course of disease” does not necessarily equate with a poor general condition but simply reflects the lack of improvement of symptoms with an average or fairly good general condition. Most of the aforementioned studies [8, 21, 22] acknowledge that decisions for testing are subjective and depend on the physicians’ experiences and attitudes.

AG, whether of viral or bacterial origin, is usually self-limiting [5]. Antibiotics are mainly recommended for severely affected patients and are most effective if given early [5, 24, 25]. “Bacterial gastroenteritis” was most frequently mentioned as main reason for antibiotic therapy in our study. We cannot judge whether this reasoning was based on laboratory results or on physicians’ experience. However, only two cases with positive stool test results for pathogens not susceptible to antibiotics were prescribed antibiotics in our study. The second most common reasoning for antibiotic treatment, namely duration of illness, was also reported by Swiss GPs in an extensive qualitative assessment [8]. A study from Poland concluded that factors associated with antibacterial drug administration included the work environment of the physician (working in large practices and hospital wards favoured antibiotic prescription compared to small practices), presence of fever, or mucus or blood in stool, age of the patient and (rural/urban) residence [26]. The presence of fever, or mucus or blood in stool could also be a factor leading to antibiotic therapy in our study as the third most frequent mentioned main reason for antibiotic prescription was suffering from a specific symptom.

Some 62% of all cases with a laboratory-confirmed *Campylobacter* infection received antibiotic treatment in our study. This finding is important in the context of antibiotic resistance development. More than half of those patients received quinolones and one-third was treated with macrolides—a finding confirming results from an earlier qualitative study among Swiss GPs [8]. Given antibiotic resistance levels for fluoroquinolones as high as 55.3% for human *Campylobacter* isolates in Switzerland in 2014 [27], these studies’ findings underscore the need for changes in prescription practise in Switzerland. A similar level of resistance (60.2%) was observed in Europe in 2014 [28]. Consequently, the European Food Safety Authority and the European Centre for Disease Prevention and Control do no longer recommend fluoroquinolones for the empirical treatment of human campylobacteriosis.

### Physicians’ case management impacts on the mandatory surveillance system

A stool test was performed only for 11.6% of patients consulting a Sentinella-physician due to AG. Of these, 19.8% (95% CI 15.1–25.6) had a positive result for a notifiable pathogen. Hence, a very small proportion of 2.3% (=11.6 × 19.8%) of AG patients consulting a Sentinella-physician were actually reportable to the mandatory reporting system. This is in line with Swiss physicians’ typical treatment pattern for AG of “wait & see”, which can be followed by a “treat & see” approach or a desirable (from the perspective of the NNSID) “test & see” or “test & treat” approach based on illness progression [8]. Considering the (main) reasons mentioned for stool testing, patients with a prolonged duration of illness and patients reporting recent travel abroad are likely overrepresented among notified cases. The proportion of patients with stool testing varies substantially between countries: it was found to be 4.3 or 9.1% in France [29], 6% in Italy [30], 7% in Ireland [31], 12% in the Netherlands [32], 19% in the US [33] and 25% in Denmark [34].

The pathogen most often identified through stool testing in this study (*Campylobacter* spp.) is also the pathogen most frequently reported to Swiss national surveillance. Norovirus, which is not notifiable in Switzerland but in several countries of the European Union, was the second most common identified pathogen.

### Mild disease with high socio-economic burden

Physicians rated the general condition of AG patients as relatively good. Nevertheless, a high proportion of 86.3% of employed patients was not able to work due to the illness. Sick leave is considerable with a median of 4 days. The risk of transmission seems to play a subordinate role as a reason for inability to work. Similar findings were reported in a French study where 79% of working patients were on sick leave for a median duration of 3 days [35]. In a Danish study, only 35% of patients with AG reported having missed work or school as a result of illness [34]. However, this Danish study was a population-based study in which only 13% of patients were seen by a physician and/or hospitalised. In our study, we did not observe a difference in time from symptom onset to consultation between employed and unemployed patients (data not shown). This indicates that the need of a medical certificate is unlikely to be a main reason for consultation.

It is well known that some pathogens causing AG are easily transmitted from human-to-human, especially viruses, and contact with diarrhoea patients has been described as a risk factor for AG previously [35, 36]. In our study, 28.6% (95% CI 24.9–32.6) of the patients had contact to other people suffering from similar signs and symptoms in the 7 days preceding symptom onset. Thus, it is possible that these patients had a common source of infection or transmitted the disease among each other.

In summary, our findings suggest that AG is a common, but generally mild disease which results, however, in a high social and economic burden. The overall financial burden due to AG (including losses in productivity) is likely a multiple of the healthcare costs estimated for Switzerland in the range of €29–45 million annually [14].

### Sentinella is invaluable to investigate current public health issues

All information for this study was derived from physicians in the Swiss Sentinel Surveillance Network. This study was specifically set up by the FOPH to clarify current epidemiological questions about gastroenteritis in Switzerland, using a national primary care sentinel surveillance platform.

We consider it a strength of the study to have obtained information on diagnosis and treatment directly from treating primary care physicians. However, the actual duration of sick leave might have been longer or shorter than reported or certified by the physician. Similarly, we could not record the overall duration of the illness as in this study we could not send out follow-up questionnaires at the end of an AG episode.

A limitation of our study is the change in sampling scheme for supplementary questionnaires for the second half of the study period, especially considering that AG is subject to seasonal variation. However, we believe that changing to full sampling and using weighted analyses to adjust for the change in sampling scheme resulted in more reliable data than continuing without changing the sampling scheme and obtaining far less supplementary questionnaires.

We expected to observe a seasonality of case reports considering the literature [4, 36], results of a previous study [8] and surveillance data [12], with a peak of AG in winter (December–March) and during summer (June–September). Instead we found a decreasing number of initial consultations per 1000 PPCs over the year which we assume is partially due to reporting fatigue of the Sentinella-physicians partaking in the study. This is supported by a survey conducted among Sentinella-physicians in which they were asked about the time required for participating in the sentinel network—in total and for the different research topics. Physicians indicated that the study on AG was comparatively time-consuming although the majority indicated that the total amount of time required for notifying was acceptable [37].

## Conclusion

Not to our complete surprise, this study has shown that acute gastroenteritis is a common disease in Switzerland with consultation frequencies comparable to influenza-like illnesses. AG presented to physicians lead to substantial sick leave in the employed, resulting in considerable socio-economic costs due to productivity loss.

Furthermore, as suspected, the study confirms that the National Notification System for Infectious Diseases captures—if at all—only a fraction of the scope of the problem (see introduction for currently notifiable diarrhoea-causing pathogens). Hence, the Swiss Sentinel Surveillance Network, Sentinella, represents a very important complementary surveillance instrument to grasp principal dynamics of infectious disease epidemiology at the primary care level.

The FOPH and the Federal Food Safety and Veterinary Office, being responsible to maintain population health and food safety in Switzerland, are currently lacking effective tools for pinpointing and a comprehensive national programme addressing the control of foodborne diseases and AG. While there are efforts to increase food safety and consumer hygiene including campaigns to increase awareness for food and kitchen hygiene among consumers in Switzerland, prevention measures to reduce contamination at food production or retail level are incomplete. Overall, there is an imbalance in national disease prevention and control efforts for AG considering that national strategies to reduce the burden of seasonal influenza—an infection with a disease burden comparable to AG—exist since many years.

## Electronic supplementary material

Below is the link to the electronic supplementary material.

Supplementary material 1 (PDF 259 kb)
