# Supplementary material for: Acute gastroenteritis in primary care: a longitudinal study in the Swiss Sentinel Surveillance Network, Sentinella
Source: Infection. 2017 Aug 4;45(6):811–24. doi: 10.1007/s15010-017-1049-5 (PMC5696444; doi:10.1007/s15010-017-1049-5)
Supplement: Supplementary file 1 — Supplementary material 1 (PDF 259 kb) [file 15010_2017_1049_MOESM1_ESM.pdf]

Acute gastroenteritis in primary care: a longitudinal study in the Swiss Sentinel Surveillance Network, Sentinella

Infection

Claudia Schmutz[1, 2]*, Philipp Justus Bless[1, 2]*, Daniel Mäusezahl[1, 2†], Marianne Jost[3], Mirjam Mäusezahl-Feuz[3], Swiss Sentinel Surveillance Network

* These authors contributed equally to this paper
† Corresponding author

[1] Swiss Tropical and Public Health Institute, Socinstrasse 57, P.O. Box, 4002 Basel, Switzerland
[2] University of Basel, Petersplatz 1, P.O. Box, 4001 Basel, Switzerland
[3] Federal Office of Public Health, Schwarzenburgstrasse 157, 3003 Bern, Switzerland

**Corresponding author:**	Dr. Daniel Mäusezahl
Address:	Swiss Tropical and Public Health Institute
	Socinstrasse 57
	P.O. Box
	4002 Basel
	Switzerland
E-mail address:	daniel.maeusezahl@swisstph.ch

DOI: 10.1007/s15010-017-1049-5

Weekly questionnaire – part on acute gastroenteritis

**SENTINELLA 2014**<br>**Meldekriterien auf der Rückseite beachten!**	Erkrankungs-meldungen<br>**Seite 2**	Arzt (Code) Woche	Formular-Nr.<br>99999

Akute Gastroenteritis

Patienten-Nummer		1	2	3	4	5	6	7	8	9	10	11	12	13	14	15	16
Geboren:	Tag																
	Monat																
	Jahr																
Geschlecht (m=1, w=2)																	
Erregernachweis im Stuhl veranlasst (ja=1, nein=2)																	
Hospitalisierung (ja=1, nein=2)																	

Sentinella – Supplementary questionnaire on acute gastroenteritis

Sentinella – Ergänzungsfragebogen – Akute Gastroenteritis

Arzt-Code: ____

Meldewoche: _____ Formularnummer: ______________ Patientennummer: ____

PATIENTENINFORMATION

1. Geburtsdatum: _____/_____/______
2. Geschlecht ☐ m ☐ w
3. Bezahlte Berufstätigkeit? ☐ ja ☐ nein

KONSULTATIONEN

4. Erstkonsultation Datum: _____/_____/20____
5. Weitere Konsultationen? Datum: _____/_____/20____ Datum: _____/_____/20____ Datum: _____/_____/20____
   ☐ keine

MANIFESTATION

6. **Beginn** der Symptome Datum: _____/_____/20____ ☐ unbekannt
7. *Bitte führen Sie* ***alle*** *Symptome auf, die seit Beginn der Erkrankung bis zur Erstkonsultation aufgetreten sind.*

Symptom	Dauer[1]	Symptom	Dauer[1]
☐ Durchfall	____ Tage	☐ Appetitlosigkeit	____ Tage
☐ mit Blutbeimengung	____ Tage	☐ Übelkeit	____ Tage
☐ mit Schleim (Mucus)	____ Tage	☐ Erbrechen	____ Tage
☐ Blähungen, Druck- und Völlegefühl	____ Tage	☐ Fieber >38°C	____ Tage
☐ krampfartige Bauchschmerzen	____ Tage	☐ andere:____________________________	____ Tage
☐ Dehydratation	____ Tage	☐ andere:____________________________	____ Tage

8. **Allgemeinzustand** (AZ) nach Einschätzung des Arztes bei Erstkonsultation: *Bitte kreisen Sie auf der Skala 1 bis 10 den Wert ein, der am besten den AZ des Patienten widerspiegelt.*

Schlechter AZ 1 - 2 - 3 - 4 - 5 - 6 - 7 - 8 - 9 - 10 Guter AZ

DIAGNOSTIK

9. **Erregernachweis** in Stuhl veranlasst? ☐ ja ☐ nein

Falls ja, a) **Entnahmedatum** der ersten Probe Datum: _____/_____/20____ ☐ unbekannt
☐ Keine Probe entnommen

b) **Hauptgrund** für die Probeentnahme: *(Bitte geben Sie nur* ***einen*** *Grund an.)*

☐ schlechter AZ	☐ Aufenthalt in Institution (präventiv-hygienische Abklärung)
☐ protrahierter Verlauf	☐ Bewohner/Patient ☐ Angestellte(r)
☐ Komorbidität	☐ berufliche Tätigkeit: welche?
☐ Ausbruchsabklärung	____
☐ Alter (Kleinkind, betagte Person)	☐ Spezifische Risikoexposition
☐ spezifisches Symptom: welches?	☐ Auslandaufenthalt vor Manifestationsbeginn
______________________________	☐ Kontakt zu anderen Erkrankten[2]
☐ anderer: ________________________	☐ Kontakt zu Tieren

c) Welche **Methode** wurde verwendet? ☐ Allg. Stuhlbakteriologie (Kultur) / Einzel-Nachweisverfahren
☐ Multiplex-PCR

d) Auf welche Erreger wurde getestet und was war das **Resultat**? *(Bitte geben Sie* ***alle*** *durchgeführten Analysen an, auch bei negativem Befund, oder legen Sie eine Kopie der Laborresultate bei.)*

Erreger	Befund pos	neg	unbekannt
☐ *Campylobacter spp.*	☐	☐	☐
☐ *Salmonella spp.*	☐	☐	☐
☐ *Shigella spp.*	☐	☐	☐
☐ EHEC (VTEC/STEC)	☐	☐	☐
☐ Noroviren	☐	☐	☐
☐ Rotaviren	☐	☐	☐
☐ andere: ________________________	☐	☐	☐
☐ andere: ________________________	☐	☐	☐

[1] Falls unbekannt, schreiben Sie bitte „N/A“ in das Feld
[2] Kontakt zu anderen an akuter Gastroenteritis Erkrankten

VERLAUF

10. **Arbeitsunfähigkeit[3]?** ☐ ja ☐ nein ☐ unbekannt

Falls ja, a) Warum? ☐ wegen Symptomen ☐ wegen Übertragungsrisiko im Beruf ☐ wegen Krankheit des Kindes

b) Wie lange? von Datum: _____/_____/20____ bis Datum: _____/_____/20____

11. **Komplikationen?** ☐ ja, welche? ______________________ ☐ nein ☐ unbekannt

12. **Hospitalisation?** ☐ ja, Datum Eintritt: _____/_____/20____ ☐ nein ☐ unbekannt
Grund? _________________________

13. **Tod?** ☐ ja, Datum: _____/_____/20____ ☐ nein

THERAPIE

14. **Antibiotikagabe?** ☐ ja, Datum Therapiebeginn: _____/_____/20____ ☐ nein

Falls ja, a) **Hauptgrund** für die Antibiotikagabe? (*Bitte geben Sie nur **einen** Grund an.)*

☐ bakteriell bedingte Gastroenteritis	☐ hohes, protrahiertes Fieber (systemische Infektion)
☐ immunsupprimierte Person	☐ Dauer der Erkrankung
☐ Polymorbidität	☐ „sicherheitshalber“
☐ schlechter AZ	☐ spezifisches Symptom, welches? _____________________
☐ fordernde Haltung des Patienten	☐ anderer: ____

b) Welches **Antibiotikum**? (*Bitte geben Sie **alle** verschriebenen Antibiotika an.)*

Stoffklasse	Name/Dosierung/Dauer
☐ Aminopenicillin	
☐ mit Clavulansäure	______________________________
☐ Cephalosporin	______________________________
☐ Chinolon	______________________________
☐ Makrolid	______________________________
☐ Metronidazol	______________________________
☐ Trimethoprim+Sulfamethoxazol	______________________________
☐ Tetrazyklin	______________________________
☐ Andere	______________________________

15. **Andere** verschriebene Therapie? ☐ ja ☐ nein

Falls ja, a) welche **Therapie**? *(Mehrere Antworten möglich):*

☐ Flüssigkeitsersatz:	☐ per oral: viel Flüssigkeit (Tee, Bouillon etc.)	☐ Antiemetika
	☐ per oral: Elektrolytlösung	☐ Analgetika
	☐ per Infusion	☐ Probiotika
☐ Antidiarrhoika		☐ Spasmolytika
☐ andere: ____		

EXPOSITION

16. **Auslandaufenthalt** (mind. 1 Übernachtung) in den 7 Tagen vor Manifestationsbeginn? ☐ ja ☐ nein ☐ unbekannt

Falls ja, a) wo (Land)? _______________________________

Aufenthalt von Datum: _____/_____/20____ bis Datum: _____/_____/20____

17. **Lebensmittel und andere Expositionen** in den 7 Tagen vor Manifestationsbeginn?

	Exposition	Ja	Nein	Pat. weiss nicht	unbekannt
a)	Geflügelfleisch konsumiert?	☐	☐	☐	☐
b)	Rohmilchprodukte konsumiert?	☐	☐	☐	☐
c)	Eier konsumiert?	☐	☐	☐	☐
d)	Mahlzeit im Restaurant / „auswärts“ ?	☐	☐	☐	☐
e)	Kontakt zu Erkrankten[4]?	☐	☐	☐	☐
f)	Berufliche Exposition? Beruf:______________________	☐	☐	☐	☐

[3] Einschliesslich Eltern, die wegen Krankheit des Kindes der Arbeit fern bleiben müssen
[4] Kontakt zu anderen an akuter Gastroenteritis Erkrankten
